# Genomic survey of TCP transcription factors in plants: Phylogenomics, evolution and their biology

**DOI:** 10.3389/fgene.2022.1060546

**Published:** 2022-11-09

**Authors:** Haiying Zhou, Delight Hwarari, Hongyu Ma, Haibin Xu, Liming Yang, Yuming Luo

**Affiliations:** ^1^ Jiangsu Key Laboratory for Eco-Agricultural Biotechnology Around Hongze Lake, Jiangsu Collaborative In-novation Center of Regional Modern Agriculture and Environmental Protection, Huaiyin Normal University, Huai’an, China; ^2^ College of Biology and the Environment, Nanjing Forestry University, Nanjing, China; ^3^ College of Plant Protection, Nanjing Agricultural University, Nanjing, China

**Keywords:** TCP transcription factors, evolutionary relationship, TCP domain, basic-helix-loop-helix structure, phylogenetic analysis

## Abstract

The TEOSINTE BRANCHED1 (TBI1), CYCLOIDEA (CYC), and PROLIFERATING CELL NUCLEAR ANTIGEN FACTORS (PCF1 and PCF2) proteins truncated as TCP transcription factors carry conserved basic-helix-loop-helix (bHLH) structure, related to DNA binding functions. Evolutionary history of the TCP genes has shown their presence in early land plants. In this paper, we performed a comparative discussion on the current knowledge of the TCP Transcription Factors in lower and higher plants: their evolutionary history based on the phylogenetics of 849 TCP proteins from 37 plant species, duplication events, and biochemical roles in some of the plants species. Phylogenetics investigations confirmed the classification of TCP TFs into Class I (the PCF1/2), and Class II (the C- clade) factors; the Class II factors were further divided into the CIN- and CYC/TB1- subclade. A trace in the evolution of the TCP Factors revealed an absence of the CYC/TB1subclade in lower plants, and an independent evolution of the CYC/TB1subclade in both eudicot and monocot species. 54% of the total duplication events analyzed were biased towards the dispersed duplication, and we concluded that dispersed duplication events contributed to the expansion of the TCP gene family. Analysis in the TCP factors functional roles confirmed their involvement in various biochemical processes which mainly included promoting cell proliferation in leaves in Class I TCPs, and cell division during plant development in Class II TCP Factors. Apart from growth and development, the TCP Factors were also shown to regulate hormonal and stress response pathways. Although this paper does not exhaust the present knowledge of the TCP Transcription Factors, it provides a base for further exploration of the gene family.

## 1 Introduction

Gene family size is a variable across different plant species, exhibiting essential functional roles for adaptation, growth and development, and speciation (Templeton, 2008). Protein classification and their coding genes involves two main approaches namely: i) generating varying family size and membership in profile-based protein databases such as the Hidden Markov Models (HMM), Pfam, InterPro, and SUPERFAMILY, and ii) categorizing these gene families based on parameter-based clustering of pairwise alignments. Gene duplication events (polyploidy), deletion, and the creation of new genes among other factors contribute to the expansion of gene family size (Flagel and Wendel, 2009; Magadum et al., 2013). As a result, genome size varies in individual plant species. The advent of gene transcriptomics has enabled the identification, study, characterization, and manipulation of numerous plant genomes and gene families (Chen et al., 2019; Li et al., 2022a; 2022b). Characteristic investigation of gene families contributes to the understanding of evolutionary relationship and functional differences (Li M. et al., 2022).

Among characterized gene families, the TEOSINTE BRANCHED1/CYCLOIDEA/-PROLIFERATING CELL FACTOR1 (TCP) gene family controls growth and development in plants; named from four unrelated proteins exhibiting diverse roles, and was first described in 1999, as a small group of plant genes encoding proteins sharing the TCP domain (Zhu, 2020). The cycloidea (CYC) from *Antirrhinum majus*, controls the floral lateral bilateral symmetry through genes differentially acting along the dorsoventral axis of the flower (Luo et al., 1996; Crawford et al., 2004; Busch et al., 2019). The Teosinte branched 1 (TB1) in *Zea mays*, encodes a protein with homology to the cycloidea gene of snapdragon (Doebley et al., 1997; Lukens and Doebley, 2001). Research has shown the CYC/TB1 genes to regulate apical dominance, repressing the growth of axillary organs, and enabling the formation of female inflorescences (Meshi and Iwabuchi, 1995; Yang et al., 2016). Lastly, the Proliferating Cell Factors 1 and 2 (PCF1/2) from *Oryza sativa* binds to the promoter region in the Proliferating Cell Nuclear Antigen (PCNA) gene (Kosugi and Ohashi, 1997). The PCF 1 and 2 are involved in the meristematic tissue-specific expression of rice PCNA gene through binding to the sites IIa and IIb, leading to the formation of either homodimer or heterodimers (Kosugi and Ohashi, 2002). The afore-mentioned genes: TB1, CYC, and PCFs are known as the TCP transcription factors (TCP TFs), characterized by the presence of a TCP domain, a 59 amino acid expanse forming a non-canonical basic-helix-loop-helix (bHLH) structure (Cubas et al., 1999). Although their ancestry remains unknown (Navaud et al., 2007), their biological roles and mode of action are conserved in plant species from the bryophytes to the angiosperms. Up to date, there have been several breakthroughs in the identification and computation of TCP TFs in numerous plant species including: *A. thaliana* (Aggarwal et al., 2010), *G. biloba* (Yu L. et al., 2022), *P. edulis* (Liu et al., 2018), *S. lycopersicum* (Parapunova et al., 2014), *L. chinense* (Hwarari et al., 2022), etc. However, in some plant orders, the TCP TFs have not yet been identified (Manassero et al., 2013). Research on the TCP gene expression have supported the biological functions of TB1, CYC, and PCF genes, tailoring the TCP domain to be involved in DNA binding activities, dimerization, and protein to protein interactions (Kosugi and Ohashi, 2002). Recent reports have shown their involvement in the regulation of biotic and abiotic stresses (Yu Z. et al., 2022; Hwarari et al., 2022).

Although the comprehension of the biochemical roles and evolution of the TCP TFs has improved in the past decade, there are still some gaps. In this article, we compared and discussed the current knowledge on the classification of TCP TFs in 37 plant species from lower plants to higher plants. We believe this paper will contribute valuable insights to the TCP gene family knowledge base. Additionally, we utilized available genomic data from current and previous research in TCP phylogenetic and evolution analysis to answer some important questions regarding the TCP gene family.

## 2 TCP conserved domains

The TCP domain is highly conserved throughout plant species, forming a bHLH secondary structure, comprising of approximately 58–62 amino acid residues involved in: DNA binding, protein to protein interactions, and protein nuclear localization. The divergent evolution of the TCP domain from the bHLH domain was by insertion of a short stretch in the basic region thereby splitting the long helix into two (Aggarwal et al., 2010). Nonetheless, the TCP domain structure has remained intact (Kosugi and Ohashi, 1997; Cubas et al., 1999). The protein structural analysis of the bHLH TFs has shown that the TCP domain is characterized by a basic residue-rich region forming a typical fold of 3 short β -strands (β1, β2, and β3) and two consecutive α-helices (α-1 and α-2) (Sun et al., 2020). However, the TCP domain is different to the bHLH domain (Carrara and Dornelas, 2021). Recent demonstrations have exhibited that the topology of the TCP domain is different from the typical bHLH structure by comparing the ß-strand conformation of the basic region in a typical TCP domain with that of a typical bHLH protein (MyoD, PDB:1mdy), and concluded that the bHLH domain of TCP conforms to a new topology distinct from a typical bHLH structure. Interestingly, their analysis in rice *OsPCF6* protein, disclosed that the TCP domain dimerize with other two TCP domains, each forming a stable conformation that adopts the ribbon-helix-helix (RHH) fold rather than the bHLH motif previously predicted. Implying that the TCP protein can also be classified into the RHH family (Liu et al., 2019; Sun et al., 2020). Although these findings are not conclusive, the homology modelling of TCP protein has also demonstrated their ability to form homodimers and/or heterodimers with other TCP proteins to bind DNA (Parapunova et al., 2014).

**FIGURE 1:**
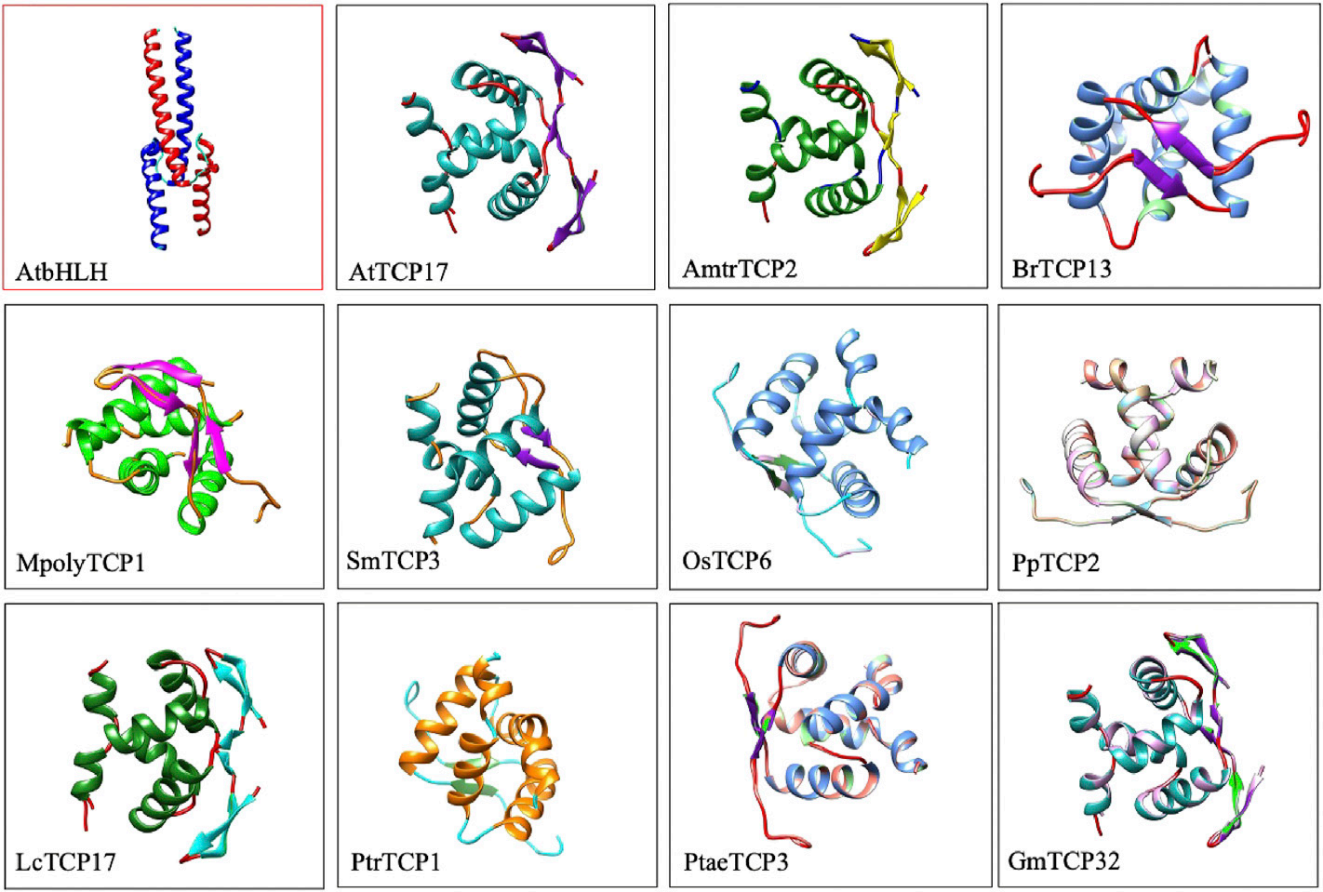
3D protein structure comparison of Arabidopsis bHLH3 against other 11 TCP proteins. Specific names of the TCP protein are highlighted below the image. 3D protein structures were searched against the online PDB database (https://www.rcsb.org/) and viewed using the chimera software.

To confirm these findings, we compared 3D protein structures of TCP proteins from 11 plants species against a single bHLH protein representative from *A. thaliana* (Figure 1). Protein structure analysis confirmed the presence of 3β-strands and 2α-helices in TCP domain, and 2β-strands and 2α-helices in the bHLH protein structure. The first and the second helices of the TCP domain were amphipathic with alternating hydrophobic and hydrophilic residues. Our analysis concurred with previous TCP protein structure investigations (Pilar Cubas et al., 1999). Recently, we have also analysed the TCP protein structure of the *L. chinense*, and showed that most of the LcTCP proteins carry 2 to 3β-strands, and 2α-helices with alternating hydrophobic regions and are less mobile (Hwarari et al., 2022). Other researches have also exhibited that the TCP protein contain potential sites of phosphorylation and regions linked by a conserved Glycine-Aspartate-Serine residues, highly frequent in loops and Proline (Cubas et al., 1999; Tarczewska and Greb-Markiewicz, 2019; Edwards and Gorelick, 2022).This result generally confirms that the TCP bHLH domain is rather distinct from the bHLH domain.

### 2.1 TCP proteins classification

**FIGURE 2:**
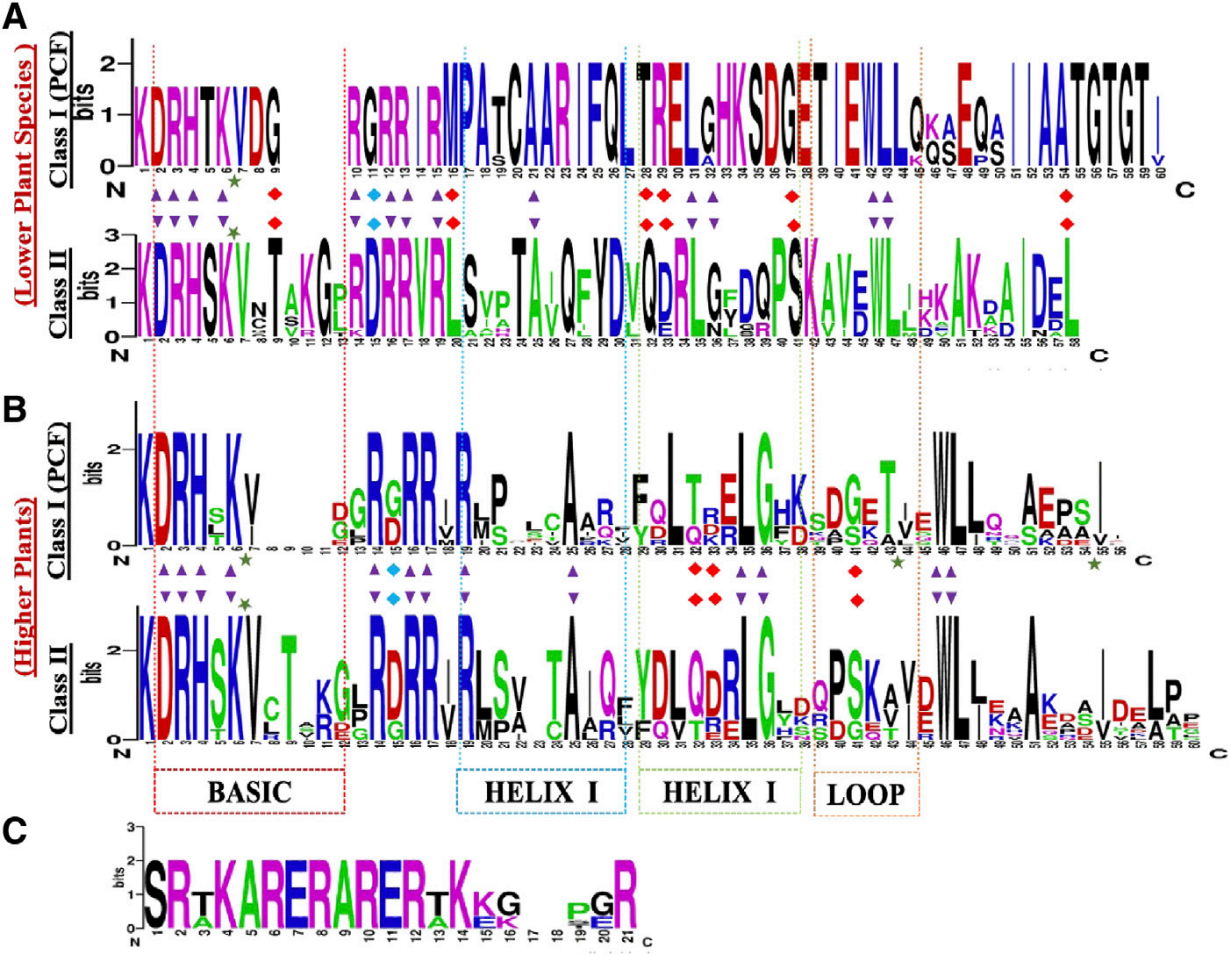
TCP protein sequence domain logos in: **(A)** lower plants **(B)** higher plants, **(C)** The angiosperm specific R-domain, present in the CYC/TB1 subclade. The sequence logos were generated by WebLogo online tool (http://weblogo.berkeley.edu/), based on the alignments of the TCP domains. The overall height of each stack letter indicates the sequence conservation at that position (measured in bits), whereas the height of the symbols within the stack reflects the relative frequency of the corresponding amino acid at that position. The black arrow depicts 90% conserved loci within the whole family. The red arrow depicts the key DNA binding site for the two subgroups. Top logo shows the TCP domain conserved within the lower plants. Bottom logo shows the TCP domain the higher plants.

The Class I TCP TFs referred to as PCF1 and PCF2, are essential for DNA binding and dimerization, they also carry the TCP domain, and are characterized by two highly conserved sequences, DRHxK and RxRRxR, in the N- and C- terminal, respectively (Figure 2) (Liu et al., 2019). Although, some class II TCP proteins lack the conserved N-terminal part in the basic region, such as the *S. lycopersicum* TCP26 (Parapunova et al., 2014), *B. rapa* TCP12a/TCP1c (Du et al., 2017). The main distinction between Class I and Class II is that, Class I has a four-amino acid deletion within the TCP domain which is absent in Class II. Deeper analysis has shown a full conservation of Class I TCP amino acids within the lower plants as compared to the higher plants (Horn et al., 2015). To confirm these findings, we constructed TCP domain logo for class I and II using the protein alignments of the TCP domain (Figures 2A,B). The TCP domain comparisons evidenced the presence of 4 amino acid deletion in angiosperms which was absent in the lower plants TCP Class I. Suggesting that the lower plants are the first forms of life or rather the extend of evolution was different between the two plant clades (Qin et al., 2021). Other researchers have also shown that the Class I TCP domain is flanked by short regions recognizing a 6–10 base pair binding sequence of GGNCCC or CCNNCC, which is absent in the Class II TCP domain (Kosugi and Ohashi, 2002). In contrast, the GGNCCC has also been shown in PCF5 of rice, a Class II member (Liu et al., 2020), which has led to conclusions that these genes share a core sequence, the GGNCCC, and they have differing flanking sequences leading to either competition or cooperation (Savadel et al., 2021).

The TCP Class II-clade, otherwise known as the CIN clade, was first isolated from snapdragon *cin*-mutant producing abnormal leaves and petals with rolled edges (Lan and Qin, 2020). Distinctively, the CIN protein carries conserved residues which are exclusive to the PCF proteins. In comparison, the Class II TCP domain has less conservation, studies have shown that the HLH region is 90% conserved, carrying Alanine (A)-25, Leucine (L)-35, (G)-36, Tryptophan (W)-46, and L-47. Additionally, there are notable distinctions in HELIX II, the first L residue has been replaced by Isoleucine (I) and Valine (V). The third L residue has also been replaced by an I residue (Martín-Trillo and Cubas, 2010; Liu et al., 2019). In total, our TCP domain comparisons, showed that lower plants Class II TCP domain is fully conserved, and exhibit significant differences in the protein sequence arrangements (Figure 2) in both the lower and higher plants.

The CYC/TB1 factors are a subdivision of Class II TCP proteins, and are angiosperm-specific. Protein structural studies of the CYC and TB1 genes have shown that they both have a 21 residue long basic region that includes a putative bi-partite nuclear localisation signal (NSL). In addition, they are characterized by the presence of an angiosperm conserved 18–20 amino acid Arginine-rich motif (the R-domain) (Figure 2C). Although, a few CIN-like proteins have also been shown to carry the R-domain (Wang et al., 2021). The R-domain forms an α-helix structure that coils similarly to leucine zippers which functions in protein-protein interaction (PPIs) mediation, and in evolutionary/developmental and phylogenetic studies (Busch and Sassone-Corsi, 1990). It is predicted to have originated independently in two separate clades, one of which is the ECE clade. The ECE denotes a conserved motif (Glu-Cys-Glu) between the TCP- and R-domain, found in most member of this clade (Smith et al., 2004).

## 3 Phylogenetics and evolution in TCP gene family

**FIGURE 3:**
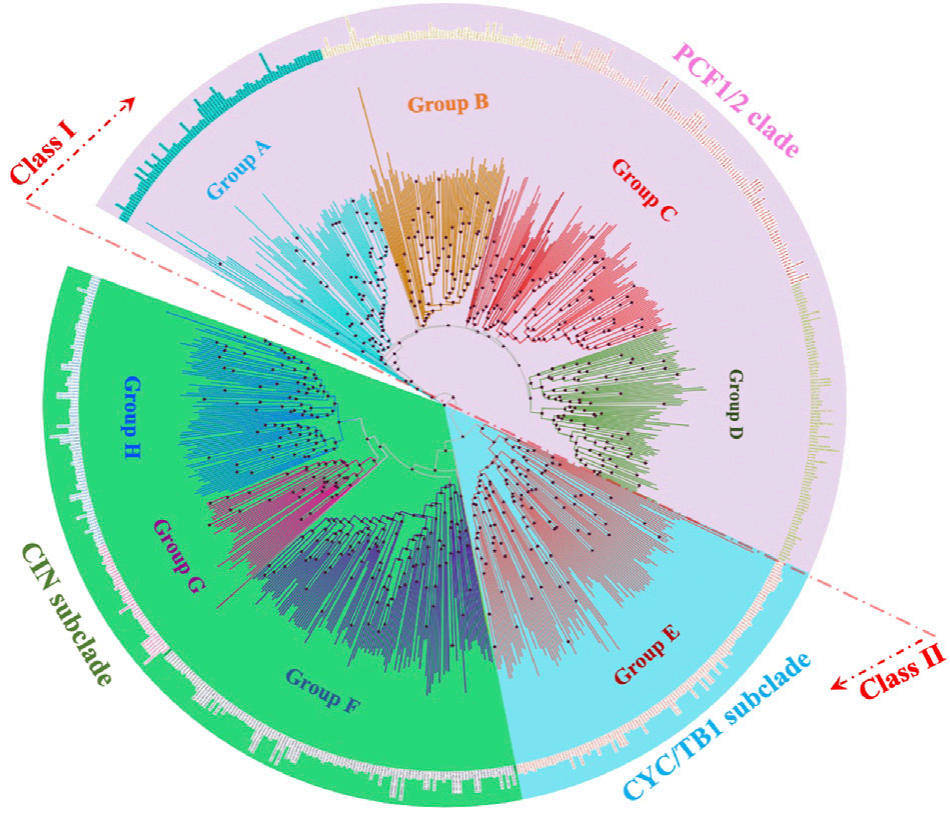
Evolutionary relationships of TCP subgroups in 28 plant species. An unrooted neighbour-joining tree was constructed with the trimmed multiple sequence alignment of MEGA 11. The phylogeny was constructed using 1,000 bootstrap replication value, bootstrap values of less than 70% are indicated by red circles on tree branches. Groups A-H were designated according to ancestral relationship of different branches and their clockwise appearance within each tree. Groups are distinguished by different branch color schemes.

System classification of the TCP gene family based on the molecular phylogeny facilitates the building of functional and genomic studies (Mondragón-Palomino and Trontin, 2011). 849 TCP proteins from 37 plant species were analyzed using the Parsimony, Maximum Likelihood (ML) and the Bayesian method. Results were consistent with corresponding values. Figure 3 shows the protein ML phylogenetic tree, our analyses concurred with previous findings that the TCP gene family can be divided into three main groups: the PCF, CIN, and the CYC/TB1 (Citerne et al., 2003). Likewise, the Bayesian inference methods have sorted TCP sequences in groups of high similarity, the class I (PCF1/2 clade) and class II (CIN and CYC/TB1) (Manassero et al., 2013; Liu et al., 2019; Yu L. et al., 2022). Nonetheless, distinctions have not been made whether the class I or class II TCP subfamily was the first to appear in plant kingdom due to the fact that lower plants believed to be first forms of plant life like *Marchantia polymorpha* carry both classes (Sharma et al., 2013). Some predictions have displayed the CIN-like TCP sub-clade in the Class II, to be more ancestral than the CYC/TB1-like TCPs since the Class II TCPs belong to the CIN-like TCP sub-clade in the non-vascular plants (Wang J. et al., 2022).

**FIGURE 4:**
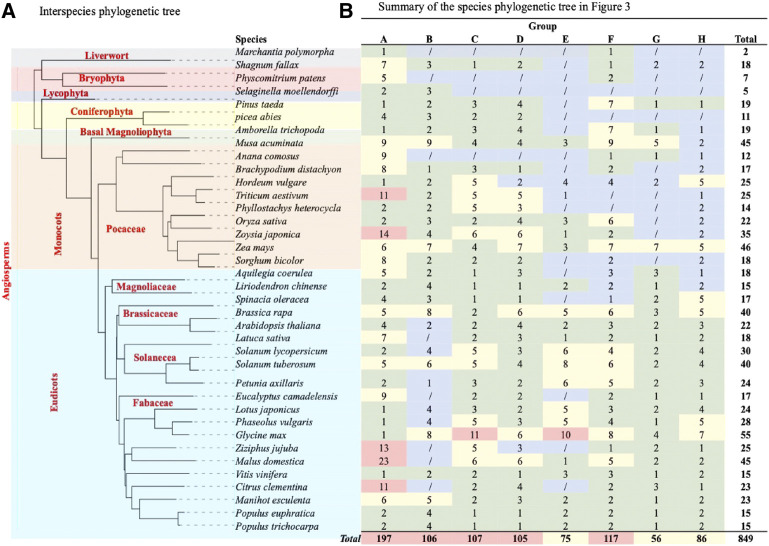
TCP phylogenetic analysis. **(A)** The interspecies phylogenetic tree constructed using Xshell ortholog finder, and the phylogenetic trees was constructed using online iTOL software. **(B)** Summary of TCP protein distribution within the phylogenetic tree shown in Figure 3. The analysed TCP proteins were grouped into eight groups (A–H) according to close ancestry and phylogeny relationships. Different color schemes represent increase in the total number of TCP proteins in specific plant species, with blue (0) to red (highest total number).

Further classifications of the TCP sequences have been shown within the phylogenetic analyses into smaller groups of high sequence similarity and possibly biological functionality. In this analysis, we divided the phylogram into eight subgroups (A-H). The PCF-clade comprised group A to D, the CYC/TB1 proteins clustered in a monophyletic group (Group E), and the CIN clade was carried in the groups F to G (Figure 4B). Group A in PCF clade had the highest number of proteins while group G in the CIN-clade had the least number of proteins. Other studies have also shown divisions of 8–10 groups depending on: the total number of sequences included in the phylogenetic research, TCP protein clustering on the same branch, and sequence structures both within and outside the TCP domain (Wang et al., 2018). We also noted that some of the plant species were fully represented in individual groups, such as *G. max* and *P. trichocarpa*, suggesting that they have undergone various gene expansion and duplications types, and also that their proteins are involved in a wide range of biological functions (Ling et al., 2020; Wang J.-L. et al., 2022). In addition, research has also shown that the TCP monocot clade can be organized into at least 20 groups, each with sequences from different species. These sequences sharing amino acid motifs extending to the TCP and carboxyl domains, and an average identity greater than 64% with the majority resembling well-supported clades of the phylogeny (Mondragón-Palomino and Trontin, 2011).

To fully understand evolution of the TCP gene family, we also analyzed the evolution of plant species in question (Figure 4A). Analysis showed that all these plants had a common ancestor, and evolutionarily events like speciation led to the formation of different plant clades and orders (Berger et al., 2016). However, monophyly of the liverwort (*M.polymorpha*) and bryophytes (*S. fallax* and *P. patens*) were shown to have diverged earlier, suggesting that these are amongst the earliest land plants (Fernandez-Pozo et al., 2022; Kumar et al., 2022). On the other hand, angiosperms diverged later into several different clades through speciation, these include the basal, magnoliid, eudicot and monocot angiosperms, possibly during the angiosperm evolution. These findings concurred with previous studies on the angiosperm species diversity and expansion (Qin et al., 2021; Hu et al., 2022).

## 4 TCP gene family duplication events

Characterization of the TCP gene family in different plant species has yielded inconsistences in the total number of TCP proteins, motif arrangement and conserved domain structures. The highest and lowest total number of TCP family members are *N. tabacum* (61) (Chen et al., 2016) and *S. officinarum* (2) according to online plant transcription factor database (PTFD; http://planttfdb.gao-lab.org/) (Tian et al., 2019). TCP genes form small families in different species which have engendered larger members of angiosperms (Li X. et al., 2022). Genome-wide searches have indicated that the expansion of the TCP gene family is by independent gene or whole-genome duplication. Lower plants, *P. patens*, *S. moellendorffii* and *M. polymorpha*, have been branded with less total number of TCP genes and none in the unicellular algae (Chlamydomonas), this may be accounted for by the fact that angiosperms have a renowned history of WGDs driven form autopolyploid and allopolyploid events (Van de Peer et al., 2009; Li X. et al., 2022). The expansion of the monocot TCP-like genes was mainly through two rounds of whole genome duplication (WGD) (Mondragón-Palomino and Trontin, 2011). Several systematic analyses of the orthologous clades from *B. distachyon, O. sativa, Z. mays* and *S. bicolor* demonstrated that their common ancestor was formed by 21 genes. These findings were also supported by other WGD research in the angiosperm genome (Landis et al., 2018; Wang et al., 2020). Although, the TCP genes have been defined as evolutionary conserved plant transcription factors grouped according to similarity, differences among them have been related to the probability of insertion or loss of introns during evolution of the species. This phenomenon may suggest that functional diversity and expression control methods have involved more replication fragments, gene doubling, and other duplication events (Li et al., 2021).

**FIGURE 5:**
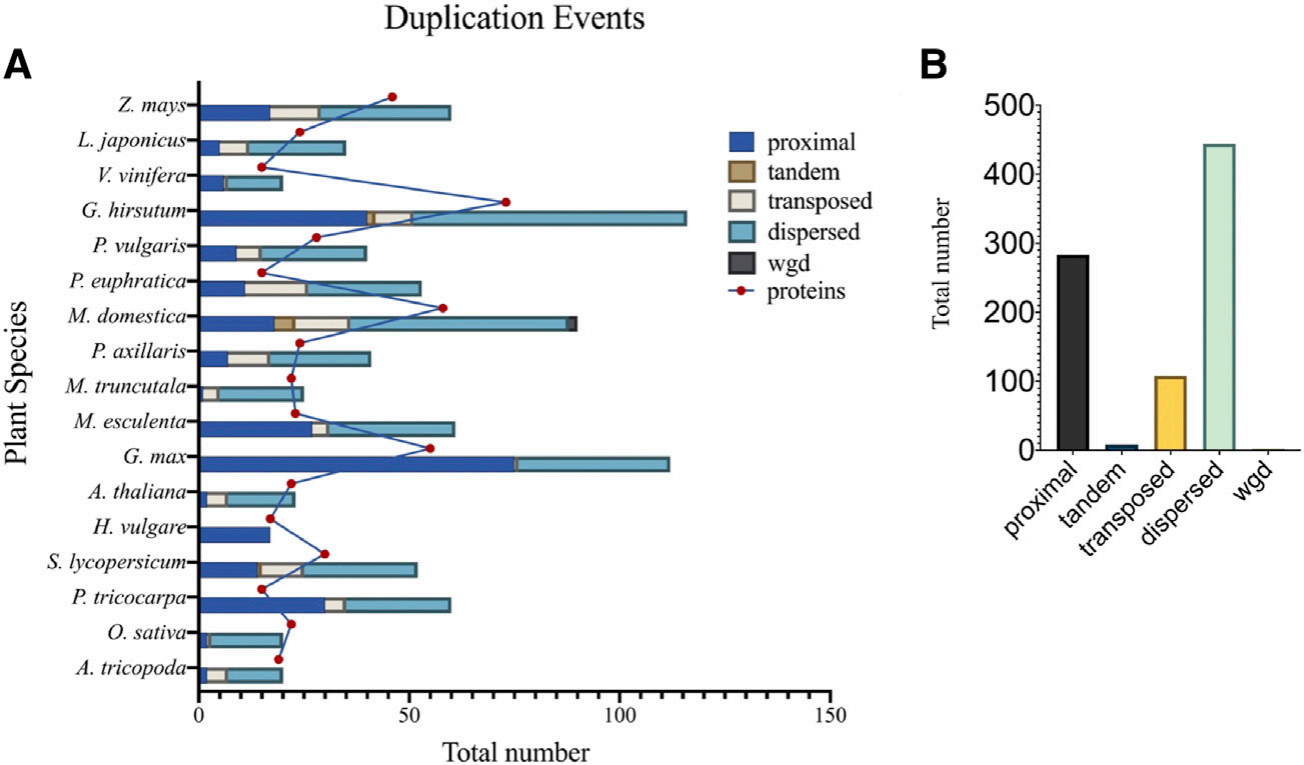
Duplication events different plant species. **(A)** Shows the number of duplication event types present in individual plant species, and **(B)** The total number of duplication events in investigated plant species. Different color backgrounds on the bar represent different gene duplication events. Also described in the key, top right corner.

Gene duplication events may take the form of segmental or single-gene duplications, involving: tandem, proximal, dispersed, and transposed duplications (Wang et al., 2012). Duplication event researches have shown that segmental duplications are the main driving force for expansion and evolution of the TCP gene family (Cannon et al., 2004; Wang et al., 2012). In support to these findings, research in Tartary buckwheat (Yang M. et al., 2022), and *M. acuminata* (Wang J. et al., 2022) have shown that the segmental duplication was responsible for the expansion of TCP gene family, and that the TCP gene family has undergone three WGDs during evolution. Therefore, to further understand the gene duplication events of TCP members in 17 different plant species, we computed for duplication event types using the plant duplicate gene database, PDGD (http://pdgd.njau.edu.cn8080) (Lee et al., 2012) (Figure 5). We observed that dispersed duplication event was the most prevalent duplication event constituting 54% of the total duplication events among the analyzed plant species, while tandem duplication events had the least prevalence of 1%. Analysis of duplicate gene pairs for each plant species showed higher percentages of dispersed duplication events in *G. hirsutum*. Tandem duplication events were only prevalent in *M. domestica* and *G. hirsutum*. Transposed duplication events were also noticed in all the plant species except in *H. vulgare* (Figure 5A). Therefore, we concluded that the dispersed duplication event contributed to a greater extend the expansion of the TCP gene family bringing about inconsistencies in the total number of TCP proteins within plant species (Figure 5B).

In addition, the CYC/TB1clade has undergone several duplications and diversifications at the base of core eudicots, giving rise to three distinct clades: CYC1, CYC2, and CYC3 (Reeves and Olmstead, 2003; Smith et al., 2004). The CYC1 is more diverse than the other two copies, containing about 8% more sequence divergence in the TCP domain (Zhao et al., 2019), and it is sister clade to the CYC2 and CYC3 clades. While, the CYC3 clade is the only clade that does not contain additional duplications from other lineages. Notably, the CYC2 exhibits the greatest number of within-clade duplication, and contains *CYCLOIDEA* and its orthologs (Howarth and Donoghue, 2006). The TB1, a single copy from *Zea mays* is more similar to genes in the CYC1 clade, compared to CYC2 and CYC3 (Doebley et al., 1997).

## 5 MicroRNAs target TCP genes and gene ontology of TCP gene family

The miRNAs control gene expression by binding to the target messenger RNA (mRNA), studies have shown the miR319 among other mRNAs to bind the TCP genes for effective regulation of their biological functions (Fang et al., 2021; Gao et al., 2022). Accumulating evidence has revealed the role of miR319-regulated TCPs (MRTCPs) in various biological pathways controlling growth and development and abiotic stress regulations (Fang et al., 2021). In *A. thaliana* the CIN-like TCP clade comprise eight members, divided into two clades based on the presence of microRNA (miRNA) binding sites outside the TCP domain. miRNA binding sites exist in *TCP2, TCP3, TCP4, TCP10,* and *TCP24* and are post-transcriptionally regulated by miR319. On the other hand, a small clade called the *TCP5*-like CIN-TCPS is formed by *TCP5, TCP13,* and *TCP17,* and it is critical in plant thermophogenesis (Han et al., 2019). Nonetheless, other miRNAs have been shown to bind TCP genes, a total of nine microRNAs have been shown to regulate twenty TCP genes in three Apiaceae species with miR319 having most target genes targeting 11 TCP genes, miR172 and miR181 targeting 3 TCP genes each. Thereby, evidencing that the miRNAs target TCP genes to execute their biological functions (Pei et al., 2021). Studies in sweet potato have identified 4 *IbTCP* genes containing miR319-bindibg sites, further investigation have confirmed that *IbmiR319* plays a crucial role in leaf anatomical morphology, and inhibits the expression levels of *IbTCP11/17* (Ren et al., 2021). The miR319 is also involved in modulating leaf morphogenesis and flowering, and the positive regulation of leaf senescence in Arabidopsis through the overexpressed *ApTCP2* influencing the JA biosynthesis (Zhu et al., 2022). The miRNA319 target 3 *TCP* genes in *C. nankingense* (*CnTCP2/4/14*). Expression analysis in *Arabidopsis* transgenic confirmed that the *CnTCP4* negatively regulates the cold stress by downregulating the cold-induced genes such as *AtCBF1/2/3*, *AtCOR15A*, and *AtKIN1*(Tian et al., 2022).

## 6 Biological functions

**FIGURE 6:**
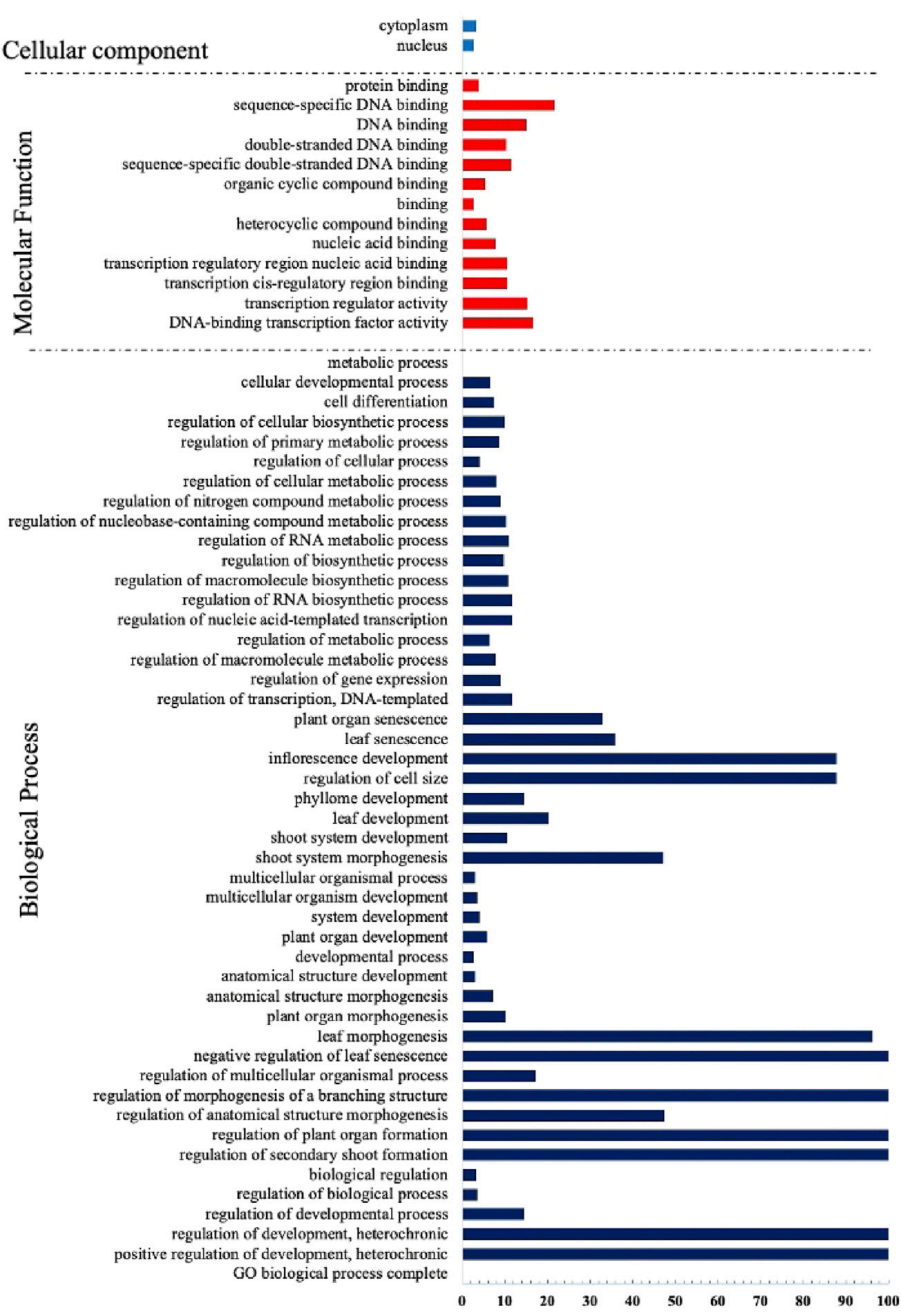
Gene Ontology Analysis of the TCP proteins in A. thaliana. GO terms were assigned using the online tool Gene Ontology Resources (http://geneontology.org/) from *A. thaliana* gene IDs.

Similar to other transcription factors, the TCP factors have undergone considerable evolutionary measures and rearrangements that created novel protein biological functions (Bornberg-Bauer and Albà, 2013). Thus, they regulate several aspects of plant development including; whole plant stature, leaf morphogenesis and maturation, inflorescence stem growth and floral organ development (Chai et al., 2017). To have an insight of the potential biological roles of the TCP genes, we performed Gene ontology analysis using *A. thaliana* TCP protein sequences (Figure 6). We observed that many processes were assigned to the biological processes (BP), mainly involved in growth and development, concurring with previous findings (İlhan et al., 2018; Kiseleva et al., 2022). A fewer processes in molecular function (MF), also had significant fold changes, and most of them were involved in DNA binding, supporting previous findings that TCPs are involved in DNA binding (Kosugi and Ohashi, 2002; He et al., 2021). The least number of processes were assigned to the cellular component (CC) category, although significant fold changes were observed. We assumed that fewer TCPs were involved with cell and cytoplasm processes. In accordance with studies in Tartary buckwheat (Yang Q. et al., 2022).

### 6.1 Class I TCP gene

Expression analysis of the Class I TCP genes in *A. thaliana* (*AtTCP15/14/8/22*) has demonstrated that the plants that lack functionality in Class I TCPs display poor stamen elongation. Suggesting that the Class I TCPs are involved in stamen elongation. For instance, *AtTCP15* regulates the expression of the Small Auxin Up RNA 63 (SAUR63) gene family, which is involved in both petal and stamen development suggesting a *AtTCP15- SAUR63* mode of action in the regulation of stamen development. Studies have also shown that *AtTCP14* and *AtTCP15* are required for optimal petiole and hypocotyl elongation during heat stress (Gastaldi et al., 2020; Ferrero et al., 2021). Overexpression analysis of Class I *GbTCP,* a homolog of *AtTCP15* yielded fewer trichomes on the stem, smaller flowers, longer pedicel, and more buds. Interestingly, the overexpression of *GbTCP* in *A. thaliana* promoted root hair initiation and elongation. Further analysis demonstrated that *GbTCP* regulates the biosynthesis of jasmonates (*LOX*_*4*_ *, AOS, AOC*_*3*_, *and OPCL*_*1*_) and other phytohormones including Ethylene and Auxin Response genes (Hao et al., 2012).

The Class I TCP transcription factors are also involved in abiotic stress regulation. The *PeTCP10* in *P. edulis* was recently shown to alleviate salt stress, and reduce cellular damage in *A. thaliana*. Additional biochemical analysis exhibited that overexpressed *PeTCP10* increases chlorophyll content, improves antioxidant capacity, and reduces water loss. Implying that the *PeTCP10* might regulate salt stress through the *CBL/SCaBP-CIPK/PKS* pathway. Additionally, *PeTCP10* was also shown to repress seedling growth and seed germination under high saline conditions (Pandey et al., 2015; Xu et al., 2021). Previous research has also heralded similar pathway in salt stress regulation by *AtMYB49* (Zhang et al., 2020), and *OsTCP19* in the *AB14*-mediated pathway (Mukhopadhyay and Tyagi, 2015). Recently, the *CmTCP9* from *C. morifolium* expressed in *A. thaliana* has enhanced the enlargement of leaves and petals, and shown to interact with multiple hormone pathway protein, such as the GA3ox4, a key enzyme in the biosynthesis of gibberellin (Yu et al.).Furthermore, the class I TCPs are involved in plant biotic stress defence. The expression patterns of *StTCP23* from Potato were analysed in susceptible variety inoculated with pathogen *Streptomyces turgidiscabies.* Their results prompted a suggestion that *StTCP23* decrease the pathogenicity of bacterial scab in Potato (Bao et al., 2019). The PIF4 transcription factors bind to the GA20ox1 and the growth regulator genes, HB1 and PRE6, for gibberellin biosynthesis (Filo et al., 2015). *AtTCP14* and *AtTCP15* were shown to significantly play a role in hormonal biosynthesis, by directly participating in the induction of HB1 and PRE6 and targeting the same genes targeted by PIF4, enhancing PIF4 binding affinity to growth regulator genes (Ferrero et al., 2019).

### 6.2 Class II TCP genes

The TCP CIN-clade is involved in cell elongation in the hypocotyl (Rath et al., 2022). In *A. thaliana TCP4* and *TCP24* suppress photomorphogenesis as compensatory measures to promote hypocotyl elongation. Analysis in the *Chirita heterotricha* CYC homologous genes, *ChCYC1C* and *ChCYC1D* has led to the conclusion that the *ChRAD* genes contain promoter binding sites for the CYC genes. Directly targeting the *ChCYC1* genes, and enhancing the floral dorsoventral asymmetry (zygomorphy) in *C. heterotricha* and other core eudicots (Yang et al., 2010; Rath et al., 2022). In *T.fournieri, TfTCP8* and *TfTCP13* genes were demonstrated to reduce the leaf breadth, affirming previous research that ectopic overexpression of *AtTCP15* a homologous gene to *TfTCP8* narrows leaf size (Huang and Irish, 2015; Zhang et al., 2021). The *OsPCF7* in rice has been shown to have a great relationship with rice tiller and heading. Comparative expression analysis between transgenic rice carrying the *OsPCF7* gene with the wild seedlings evidenced its functional roles in increasing shoot height, root length, and grain yield. Deeper analysis in the mode of function demonstrated that the *OsPCF7* increased the expression of downstream genes, the Class I *KNOX* genes such as: *STM, KNAT*_*2*_ *, KNAT*_*6*_ *, LOX*_*2*_ *, AS1* and indoleacetic acid-induced protein_3_ (*IAA*_*3*_ *)* (Li et al., 2020).

Comparative expression analysis of the CIN-clade in *Antirrhinum majus* between wild type and the CIN-mutant genes, has shown that the CIN-clade inhibits excess cell proliferation, maintaining the leaf surface flatness by regulating its phytohormonal pathways. Molecular analysis revealed that CIN-clade TCPs directly bind to genomic regions promoting the transcription of cytokinin receptor homolog HISTININE KINASE 4 (AmHK4) and INDOLE-3-ACETIC ACID INDUCIBLE3/SHORT HYPOCOTYL 2 (IAA3/SHY2) (Das Gupta et al., 2014). Ectopic expression of *G. raimondii TCP11* in *A. thaliana* has shown that *GrTCp11* suppresses JA and Ethylene biosynthesis pathways thereby, reducing root hair elongation; through targeting genes that are directly associated with *AtLOX4, AtAOC3, AtJAZ1, AtJAZ2,* and *AtMYC2* (Hao et al., 2021).

#### 6.2.1 TB1 genes

The TB1 subfamily regulates branching in various plant species (Aguilar-Martínez et al., 2007). Expression analysis of *G. hirsutum TCP62* have proved these findings, and showed a high enrichment in the auxiliary buds and phyllophores. Nonetheless, overexpression of *GhTCP62* in *A. thaliana* has shown a negative regulation of total number of shoots and reduced growth vigor (Liu J. et al., 2021). The Branched and Indeterminate spikelet 1 (BDI1), which encodes a TCP transcription factor, and highly conserved in both wild and cultivated barley has been shown to play a crucial role in determining barley inflorescence architecture and spikelet development; through regulating the gene transcription of cell wall modification and known Trehalose-6-phosphate homeostasis (Shang et al., 2020). The COMPUSITUM1 (COM1) in Class II CYC/TB1 subclade, working independent of the COM2 has been shown to inhibit spike-branching through boundary defined signals linked to the SM identity pathway, *VRS4(HRA2)—COM1- HvLG1* (Poursarebani et al., 2020)*.* Ranunculales, a sister order to three eudicots*, P. somniferum, E. californica,* and *C. vesicaria,* has been shown to play a crucial role with a wide diversity in developmental traits through the expression of CYL genes (Zhao et al., 2018). Although, the mode of action still requires further research. The overexpression of *V. vaccinium TCP18* has been demonstrated to significantly decrease seed germination which can be alleviated by stratification and low temperature regulation, through a negative feedback loop. *VvTCP18* is downregulated by low temperatures further preventing its binding to the FT, thereby retaining the normal function of the FT (Maurya et al., 2020; Li et al., 2021). Additional regulatory roles of theTCP gene members from various plant species are summarized in Table 1.

**TABLE 1:** Summary of some genes and their biological functions

Plant species	Gene ID	Transgenic plant	Function	Reference
*P. TRICHOCARPA*	*PtrTCP10*	*P. trichocarpa*	Salt stress	Wang et al. (2022c)
*G. BARBADENSE*	*GhTCP*	*A. thaliana*	root hair initiation and elongation	Hao et al. (2012)
T. F*OURNIERI*	*TfTCP8/13*	*A. thaliana*	Leaf and flower shape	Zhang et al. (2021)
*G. HIRSUTUM*	*GhTCP62*	*G. hirsutum*	Regulates branching	Liu et al. (2021b)
		*A.thaliana*	Regulates shoot growth vigor	
*O.SATIVA*	*OsPCF7*	*O.sativa*	Regulates rice grain yield	Li et al. (2020)
*P. eDULIS*	*PeTCP10*	*A.thaliana*	Regulates salt stress	Chen et al. (2019)
			Inhibits seed germination and seedling under salt stress	
*C. NANKINGENSE*	*CnTCP4*	*A.thaliana*	Inhibits cold-inducible gene expression	Tian et al. (2022)
	*CnTCP9*	*A.thaliana*	Leaf development	(Yu et al., 2022b)
		*C. morifolium*	Flower enlargement	
*C. PUMILA*	*CpCYC*	*C. pumila*	Floral zygomorphy, horizontal orientation of flowers, dorsal petal and lateral staminodes orientation	Liu et al. (2021a)
*Z. JUJUBE*	*ZjTCP16*	*A. thaliana*	Leaf morphogenesis	Yang et al. (2022b)
		*Z. jujuba*	Cell proliferation	
*H. VULGARE*	*BDI1*	*H. vulgare*	Inflorescence architecture	Shang et al. (2020)
	*COM1*	*T. aestivum L*	Spikelet development	(Poursarebani et al., 2020)
			Inhibits spikelet development	
*P. SOMNIFERUM*	*EsaCYL1/2*	*E. californica*	Axillary shoot branching	Zhao et al. (2018)
		*C. vesicaria*	Regulation of petal size and stamen number	
			Affect floral symmetry	
*V. VACCINIUM*	*VcTCP18*	*A. thaliana*	Seed germination rate	Li et al. (2021)
*B.PAPYRIFERA*	*BpTCP8/9/14*	*A. thaliana*	Prevent rosette branch outgrowth	Zhao et al. (2020)
*D. LATIFLORUS*	*DlTC12-C*	*D. latiflorus*	Inhibits lateral branch growth	Jin et al. (2022)
*G. RAIMONDII*	*GrTCP11*	*A. thaliana*	Inhibits root hair elongation	Hao et al. (2021)
			Suppresses JA and Ethylene pathways	
*A. PALMATUM*	*ApTCP2*	*A. thaliana*	Modulate leaf morphogenesis	Zhu et al. (2022)
			Affect flowering	
			Positively regulate leaf senescence	
*M. POLYMORPHA*	*MpTCP1*		Controls cell proliferation and redox processes	Busch et al. (2019)

## 7 Conclusion and perspectives

Characterization and expression research of the TCP TFs has progressed quite well in the past decades, and has improved the understanding of the TCP gene family. In this article, we summarized recent findings and answered a few questions in regard to the TCP gene family through phylogenetic and duplication analysis. TCP factors have been classified into the PCF-, CIN-, and CYC/TB1 clades. This diversity has brought a wide range of biological functionality in hormonal, growth and development, biotic and abiotic, and other numerous biological processes. Implying a crucial role within the TCP gene family. Biomolecular studies have also revealed the basis of functionality of the TCPs, which is the bHLH domain responsible for DNA-binding and protein to protein interaction. Suggesting that TCPs can bind to other proteins or DNA to effectively perform their biological roles. Although, the TCP TFs may carry similar TCP domain in different plant species, studies have also revealed inconsistencies in the TCP gene family size amongst plant species. This phenomenon can be related to duplication and deletion events of plant genome that contribute to the expansion of gene families. Several duplication events have been discussed and shown to have contributed to the overall expansion of the gene family notably, here we concluded that the dispersed duplication event contributed to a greater extend in the investigated plant species. Evolution can also be accounted for in plant diversity, we investigated the evolution of TCP genes, basically we noted that the PCF-clade was fully conserved in bryophytes, lycophytes and liverworts as compared to the angiosperms, and that lower plants lacks the R-domain in the CYC/TB1 subclade. This we related to the fact that the angiosperm evolution brought about increased speciation and probably deletions within the TCP conserved motifs and domain. Nonetheless, TCP genes in other plants are still yet to be characterized and their substantial functional roles elucidated.

Future characterizations of TCP gene family should provide resources for plant genetic improvements, offer directions for practical use, and fully disclose the regulatory mechanism by which the TCP genes control abiotic stress response, and growth and development through genetic transformation or gene editing (Min et al., 2022). In addition, future TCP protein to protein network studies should map different pathways that interact with the other proteins. In conclusion, cumulative knowledge gained from these summarized studies will generate novel morphologies of agronomic interests and help bio-engineer enhanced resistant plants to environmental stress and pathogens.
